# The relationship between omega-3 and smoking habit: a cross-sectional study

**DOI:** 10.1186/s12944-016-0220-9

**Published:** 2016-03-22

**Authors:** Nóris Scaglia, José Chatkin, Kenneth R. Chapman, Ivone Ferreira, Mario Wagner, Peter Selby, Johane Allard, Noe Zamel

**Affiliations:** Pontifícia Universidade Catolica do Rio Grande do Sul (PUCRS), Porto Alegre, Brazil; Asthma & Airway Centre, University Health Network, Toronto, Canada; Asthma and Airways Centre, McMaster University, Toronto, Canada; Department of Family and Community Medicine and Psychiatry, University of Toronto, Toronto, Canada; Department of Medicine, Toronto General Hospital, University of Toronto, Toronto, Canada; Department of Medicine, Division of Respiratory Diseases, University of Toronto, Toronto, Canada; Hospital São Lucas da PUCRS – Centro Clínico, 6690 Ipiranga Ave, Porto Alegre, RS 90610000 Brazil

**Keywords:** Omega-3, Cigarette smoking, Eicosapentaenoic acid, Docosapentaenoic acid, Fatty acids

## Abstract

**Background:**

Omega3 polyunsaturated fatty acids (PUFAs) are related to several diseases, including smoking. The aim of this study was to evaluate the relationship between omega-3 intake and tobacco smoking, taking into account the qualitative differences in dietary intake between smokers and non-smokers, the amount of the ingested PUFA and their red blood (RBC) contents. We also looked for an association between omega-3 RBC content and smoking, and also between omega3 intake and the level of nicotine dependence.

**Methods:**

Using a cross-sectional study, we included 50 current smokers (group I) and 50 lifetime non-smokers (group II), aged 18–75 years. We screened them at the Toronto Western Hospital and the Centre for Addiction and Mental Health (Toronto, Canada). The subjects completed a questionnaire with demographic data, lifestyle habits and details of food intake. The PUFAs measured in the RBC membranes were alphalinolenic acid, eicosapentaenoic acid (EPA), docosapentaenoic acid and docosahexaenoic acid (DHA). In order to perform an adjusted comparison between smokers and non-smokers we used the ANCOVA model.

**Results:**

After adjusting for confounding factors, non-smokers showed higher consumption of PUFAs, especially salmon: 800 g (0–7.740) than smokers 430 g (0–2.150) *P* < 0.001. They also had higher DHA levels compared to smokers: 4.81 % (2.79–10.21) and 4.13 % (2.33–7.73), respectively, *p* < 0.05. The other PUFAs showed no significant differences between the two groups.

**Conclusions:**

Smokers ate less fish rich in omega3 fatty acids than non-smokers, showing and inverse and significant relationship between omega3 intake and smoking. Smokers had lower levels of DHA and EPA, a not previously reported finding. Considering that PUFAs probably interfere in smoking habit, the increase in omega-3 consumption may become a perspective in prevention or treatment of smoking. However, this inference must be evaluated through specific studies.

## Background

The omega-3 fatty acids are important components of cell membranes and have a role in numerous human metabolic processes [1]. These substances may contribute to the prevention and treatment of neurological [2, 3] and cardiovascular diseases [4–6], many chronic inflammatory and respiratory diseases [7–10], several of which are also linked to tobacco smoking [11–13].

Omega-3 intake has been linked to positive effects on brain functioning, affecting multiple processes in neurotransmitter pathways, synaptic transmission, membrane permeability and signal transduction [14, 15].. On the other hand, low levels are related to several neuro-psychiatric diseases such as attention deficit hyperactivity disorder, depression, schizophrenia [16–19], Alzheimer’s disease [2].

Smoking may affect omega-3 levels and PUFAs (Polyunsaturated Fatty Acids) are also considered to have a role also in tobacco addiction. Such dual interference is related to various mechanisms. [17] Smoking can increase the lipid peroxidation of PUFAs by promoting oxidative stress [20, 21]. Another possibility is that tobacco smoke may affect the synthesis and metabolism of fatty acids, and thus modifying the omega-3 levels. Smoking may also be associated with a reduced dietary intake of omega-3 due to unhealthy lifestyle, including changes in dietary food choices associated. A taste change among smokers is also another possibility [6, 18–20]. However, differences in omega-3 dietary intake between smokers and non-smokers have not been fully studied, and the relationships have not been thoroughly analyzed [6, 11, 15, 18–23].

Despite the importance of these substances in the human diet, there is no consensus in the literature regarding optimal omega-3 levels. The omega-3 index is considered a marker of PUFA status. It represents the sum of the percentages of eicosapentaenoic (EPA) and docosahexaenoic (DHA) acids measured in the red blood cell membrane (RBC). It is expressed as a percentage of total erythrocyte fatty acids content. The recommended value of 8 % or greater of the total PUFAs probably prevents coronary heart disease in Western populations. However, if the index value is lower than 4 %, there is an increased risk of mortality due to cardiovascular events [24, 25]. Its role has not been studied in other diseases.

Moreover, there is initial evidence that the usual diet of smokers and non-smokers is different, especially in relation to the consumption of fish and other foods rich in omega-3 [6, 18, 20, 26].

Therefore, we decided to study the relationship between smoking and possible qualitative differences in dietary intake between smokers and non-smokers, especially consumption of omega-3, taking into account the source and the amount of food ingested. The objective was to evaluate the relationship between the intake of foods rich in omega-3 among smoker and nonsmoker volunteers. In addition, we searched for an association between RBC omega-3 content and smoking load history and the level of nicotine dependence.

## Results

We screened 112 patients, but 6 subjects in each group did not satisfy all the selection criteria (6 due to be using omega-3 supplements, 3 due currently unstable diseases, 2 due a strict vegetarian diet and 1 due inability to understand the study procedures). Therefore, we included 50 volunteers in each group (smokers-group I and nonsmokers-group II).

Table 1 presents the basic characteristics and habits of the included subjects. Smokers were older than non-smokers (53.0 ± 13.3 years vs. 40.2 ± 13.6 years, respectively; *P* < 0.001) and had a significantly higher BMI than non-smokers (27.2 ± 5.4 kg/m^2^ vs. 24.2 ± 3.7 kg/m^2^, respectively; *P* = 0.002). There were no significant differences between the two groups in gender, education level, regular physical activity or weekly alcohol consumption.

**Table 1:** Characteristics and habits of the volunteers

Variable	Smokers *n* = 50	Nonsmokers *n* = 50	*P*
Age, years	53.0 (±13.3)	40.2 (±13.6)	<0.001^*^
Female sex, n^o^ (%)	25 (50.0)	31 (62.0)	0.31^#^
Education ≥ 9 years, n^o^. (%)	36 (72.0)	39 (78.0)	0.65^#^
BMI, kg/m^2^	27.2 (±5.4)	24.2 (±3.7)	<0.002^*^
Physical activity, n^o^sessions/week	0.0 (0 to 7)	2.3 (0 to 7)	0.81’
Food consumption, grams/month			
Fish	430 (0 to 2150)	800 (0 to 7740)	<0.001’
Shrimp	50 (0 to 1290)	100 (0 to 1500)	0.13’
Squid	0.0 (0 to 100)	0.0 (0 to 875)	0.11’
Sushi	0.0 (0 to 1720)	100 (0 to 2500)	0.060’
Flaxseed	0.0 (0 to 172)	0.0 (0 to 194)	0.68’
Walnuts	0.0 (0 to 455)	0.0 (0 to 645)	0.33’
Other nuts	25 (0 to 4515)	129 (0 to 8600)	0.13’
Granola	0.0 (0 to 1505)	0.0 (0 to 6020)	0.96’
Alcohol consumption	130 (407 to 1050)	136 (407 to 568)	0.34’
Pack-years	33.8 (±30)	-	-
Nicotine dependence (Fagerstrom test)	5.36 (±2,97)	-	-

Data are presented as counts (percentages), mean (±SD) or median (minimum to maximum)

*Student’s t test; #chi-square test; ’Mann-Whitney U test

The median (minimum and maximum) monthly consumption of fish was significantly lower in smokers than in non-smokers: 430 g (0–2.150) vs. 800 g (0–7.740), respectively; *P* < 0.001.

Group I subjects smoked a mean (± SD) of 33.8 ± 30 pack-years and had a nicotine dependence of 5,36 (±2,97), according to the Fagerstrom test for nicotine dependence (FTND) [27].

Table 2 presents the percentage (%) of each omega-3 fatty acid in the RBC membranes measured in this study in relation to the total fatty acid content. The values represent the medians (minimum to maximum values). Only DHA was significantly different in the two groups, being 4.13 % (2.33–7.73) and 4.81 % (2.79–10.21), *P* = 0.005 for smokers and non-smokers, respectively. In all the remaining PUFAs assessed (alpha-linolenic, eicosapentaenoic, docosahexaenoic acids) no significant differences between groups were detected.

**Table 2:** RBC membrane omega-3 fatty acids percentage in subjects of the sample

Acid Coding	Fatty acid nomenclature	Smokers *n* = 50	Nonsmokers *n* = 50	*P*
18:3	Alpha-linolenic	0.32 (0.00 to 0.85)	0.26 (0.00 to 0.66)	0.43
20:5	Eicosapentaenoic	0.95 (0.35 to 2.24)	1.06 (0.13 to 2.93)	0.40
22:5	Docosapentaenoic	2.29 (1.43 to 3.45)	2.26 (1.64 to 3.17)	0.71
22:6	Docosahexaenoic	4.13 (2.33 to 7.73)	4.81 (2.79 to 10.21)	0.005

Data presented as median (minimum to maximum); measurement unit as the percentage (%) of each acid related to the total fatty acids. *RBC* red blood cell

To evaluate the relationship between fish intake by month and smoking, we carried out univariate and multivariate analyses, as shown in Table 3. In the univariate analysis, the geometric mean ratio (GMR) between non-smokers and smokers was 2.16 (95 % CI: 1.36–3.45); *P* = 0.001, η^2^ = 0.10. After adjusting for possible confounding factors such as age, gender, education, BMI, consumption of other foods and alcohol and physical activity, the GMR changed to 2.03 (95 % CI 1.09–3.78); *P* = 0.026 and η^2^ = 0.07.

**Table 3:** Fish consumption: Geometric Mean Ratio between smokers and nonsmokers about (in grams per month)

Model	Smokers	Nonsmokers	GMR	95 % CI	*P*	η^2^
	*n* = 50	*N* = 50				
Unadjusted	276	598	2.16	1.36 to 3.45	0.001	0.10
Ajusted*	333	676	2.03	1.09 to 3.78	0.026	0.07

Data described as geometric mean; GMR: geometric mean ratio of non-smokers to smokers representing the relative difference or fold change; η^2^: Eta square (ANCOVA’s measure of effect size); *variables included in the model were age, sex, education, BMI, alcohol intake, and regular weekly physical activity

There was a significant difference in the consumption of fish among the participants of the two groups. Regular intake of salmon was reported by 28 % of the smokers and by 52 % of the non-smokers (*P* = 0.025). The difference in consumption of the other types of fish was not significantly different between the groups (Table 4).

**Table 4:** Fish consumption rates in smokers and non-smokers

Fish	Smokers *n* = 50	Nonsmokers *n* = 50	*P*
Only salmon	14 (28.0)	26 (52.0)	0.025
Trout	2 (4.0)	5 (10.0)	0.44
Tuna	8 (16.0)	8 (16.0)	0.99
Haddock	2 (4.0)	3 (6.0)	0.88
Tilapia	3 (6.0)	4 (8.0)	0.91
Others	7 (14.0)	16 (32.0)	0.057
>1 type	14 (28.0)	15 (30.0)	0.99

Data presented as counts of subjects: n (%)

The relationship between monthly fish intake and smoking load was not significant, using pack-years as a marker. The correlation coefficient was r_s_ = 0.05; *P* = 0.74. Likewise, the relationship between monthly fish intake and levels of nicotine dependence according to the FTND was also not statistically significant. The correlation coefficient was r_s_ = 0.01; *P* = 0.98.

## Discussion

In this study, we report that non-smokers consumed significantly more fish than smokers, especially salmon. They also had significantly higher DHA concentrations. These results remained significant after adjusting for possible confounding factors.

The role of omega-3 in smoking addiction has been previously shown by the lower omega-3 fatty acids levels among smokers [6, 7, 21, 28]. Also, the dietary supplementation of these substances has been reported as reducing tobacco cravings in regular smokers comparing with placebo treatments [11].

However, most of these studies did not take into account possible qualitative differences in dietary intake between smokers and non-smokers. Our analysis of food consumption was done assessing the feeding routine in details for each participant. We observed that non-smokers consumed significantly more salmon than smokers, a difference not seen in the intake of other fish species here studied. This finding is consistent with the higher concentrations of RBC omega-3 detected in non-smokers as the amounts of EPA and DHA in salmon are recognized to be very high.

There was no significant difference in the intake of other foods rich in alpha-linolenic acid, as shrimp, squid, sushi, flaxseed, nuts, oilseeds and granola. We speculate that these foods are typically consumed in smaller quantities and less frequently.

There are several possible explanations for the lower omega-3 content in smokers. Differences in eating choices may be a consequence of changes in the taste of foods caused by existing substances in cigarette smoke. Geographical and cultural patterns could induce a routinely different intake of foods rich in omega-3. A dietary deficiency of omega-3 may be associated with an increased risk of smoking [13].

The role of the food chain in the intricate neurobiological mechanisms related to the release of many neurotransmitters in the brain, including those related to smoking still needs to be further assessed [15, 22, 29–31]. However, it is possible that the release of specific neurotransmitters, particularly those from the dopaminergic route, caused by smoking or diet choices might interfere with food alternatives or even inducing the smoking habit [15, 31].. The low omega-3 intake could be either a risk factor for or a consequence of tobacco use [14, 15, 26]. Furthermore, the supplementation of omega-3 in these subjects may result in the renormalization of the dopaminergic system and also reducing the negative symptoms of nicotine withdrawal, but this perspective needs to be further studied.

Another possibility to explain the lower omega-3 content among smokers might be related to the metabolism of these substances. The interference of smoking with the absorption, metabolism and synthesis of fatty acids is a possibility that should be considered [28]. Also, it is known that PUFAs are susceptible to oxidation and cigarette smoke has a potent oxidant role, another proposed explanation for these findings [6, 19].

The effect of DHA in neural plasticity and in cognition mechanisms is becoming clearer. There is evidence that DHA may improve cognitive ability and facilitate synaptic plasticity [15]. The improvement of such abilities is linked to a decreased risk of neurological and psychiatric illnesses, another possible link to the inverse relationship between smoking and omega-3 intake [32, 33].

As a consequence of any one of these mechanisms, the lower concentrations of omega-3 PUFAs could affect neurotransmission in several ways in the central nervous system. The ultimate result would be an affected reward response and nicotine dependence, increasing cigarette cravings and frustrating smoking cessation attempts.

In accordance with such a possibility, a previous study has shown a daily supplement of EPA and DHA for smokers has been accompanied by a significant decrease in the number of daily cigarettes smoked and in tobacco craving episodes [11]. However, in another trial, DHA supplementation given to a small number of smokers for only a few weeks, in a low dosage, without a control group did not show reduction in the number of smoked cigarettes during the treatment period [19].

There is a group of foods that brings health benefits beyond the traditional nutritional functions. They are now labeled as functional foods. When a product is purified from foods and sold as in medicinal forms for health benefits it is called nutraceuticals. Under this perspective fish oils and omega-3 are under research and several papers have been published demonstrating their efficacy in protecting cardiovascular system and thus reducing the risk of many cardiovascular diseases [31].

However, such relationship between the nutraceutical omega-3 and smoking status is only beginning to be examined. In the present study, we detected a small, but significant difference in the omega-3 index between the group of smokers and non-smokers. The importance of this finding is unknown at this moment and needs to better evaluation.

Some limitations to our study should be noted. The major one is that, as a cross-sectional study, it does not provide any indication of the sequence of events, whether exposure occurred before, after or during the onset of the outcome. Second, we recruited control subjects from the general population via press advertisements. However, they were all Caucasian and lived in Toronto, Canada. Anyway we realize that these individuals might have different lifestyles compared to the individuals in our smoking group. Nonetheless, the reported relationships between smoking and lower DHA concentrations persisted, even after the adjustment modelling. Third, since we gathered dietary data via self-reported questionnaires and we did not verify the consumption directly, recall bias may have occurred. Fourth, we examined only Caucasian volunteers, which limit the generalizability of our results to other population groups that typically eat more fish, such as Asian populations [34, 35].

Although the sample seems small, statistical calculation was previously performed to be enough to answer the study questions, having 90 % statistical power and effect size ≥ 0.5 with significance level α = 0.05. Anyway, probably it would be necessary a larger sample to study the relationship between omega-3 RBC content and tobacco load expressed as pack-years. We detected an inverse trend, but not statistically significant, that is, the more the subject smoked, the lower was his or her omega-3 content. Similarly, not significant findings were detected in the relationship of monthly fish intake and the level of nicotine dependence, as measured by the Fagerstrom test.

## Conclusions

Our results add some aspects to clarify the importance of omega-3 intake in smoking behavior. The finding that smokers consume less fish rich in omega-3 fatty acids compared to nonsmokers is in agreement with their lower levels of DHA and EPA. We believe that these findings adjusting for food intake have not been yet demonstrated nor the intake qualitative differences. Omega-3 studies that include smokers and non-smokers must consider differences in unsaturated fatty acids intake. Future studies will be needed to determine the reason(s) for these dietary differences.

Considering that the food intake pattern is a changeable behavior, it is possible that an increase in fish consumption or DHA supplementation may have a role in smoking prevention or as an adjuvant in treatment of tobacco dependence [10]. We believe that this hypothesis deserves further studies.

## Methods

Using a cross-sectional design, we recruited current smokers, trying to quit (group I) screened at the Toronto Western Hospital and at the Centre for Addiction and Mental Health, both in Toronto, Canada. Non-smokers (group II) were recruited from the local community by press advertising. These subjects were included in the study from January to December 2013.

Caucasian men and women, aged 18–75 years were eligible to participate if they agreed to the study procedures. Exclusion criteria were refusal to sign the consent form, use of omega-3 supplements, illiteracy, inability to understand the study procedures, a strict vegetarian diet, pregnancy or nursing mothers, drug abuse, currently unstable diseases, including mental illnesses and any regular pharmacological treatment.

At the first interview, the participants were informed about the objectives and procedures of study and signed a written consent form. A trained registered nurse (author NS) interviewed and examined all participants at the same time of the day (early morning, around 8:00 AM).

Information related to demographic data, education level, alcohol consumption and level of physical activity via self-reports were collect. The subjects were asked to complete a part of a previously validated Food Frequency Questionnaire [36]. Such section of the questionnaire contains specific questions about intake of fish and seafood in general and also about the intake of other foods rich in omega-3.

Patients filled out the questionnaire considering a monthly food record frequency. In order to facilitate the identification of the size of the food portions, photos and replicas of the foods and dishes were shown to the participants.

Lifestyle habits data (alcohol intake and physical activity) were also obtained at the same interview, as possible confounding factors [20, 28]. The volunteers were categorized as alcohol non-drinkers (<180 g/week) or drinkers (≥180 g/week) [28]. We considered regular physical activity when the volunteer performed at least a ≥ 30 min session per week.

Regarding smoking, the subjects were classified into two groups: current smokers and non-smokers. Current smokers were individuals who had smoked ≥100 cigarettes in their lifetime and were still smoking daily or most days. Subjects who had smoked < 100 cigarettes in their lifetime were excluded. Non-smokers were individuals who have never smoked. Among the smokers, the exposure was quantified in pack years, where one pack year is 20 cigarettes smoked/day for one year. It was calculated multiplying the number of cigarette packs smoked per day by the number of years the person smoked [37]. All smoking patients also performed the Fagerström Test for Nicotine Dependence (FTND) [27].

Height and weight were recorded and a blood sample of the fasting patient was drawn and conducted around the same time for all participants (early morning, around 8:00 AM).

The American Heart Association recommends the consumption of two servings of fish per week for persons with no history of coronary heart disease and at least one serving of fish daily for those with known coronary heart disease, assuming that a serving size is approximately 99 grams [5].

The PUFAs here studied (alpha-linolenic, eicosapentaenoic, docosapentaenoic and docosahexaenoic acids) were measured in the RBC membranes and the levels may vary with diet and be altered by specific supplementation [26].. So, the blood sample was collected early in the morning in fasting patients. The lab tests were carried out at the Laboratory of Clinical Analysis of the University of Guelph, in the city of Guelph, Canada*.* Blood samples were taken in 4 ml test tubes containing EDTA and were centrifuged (910G for 10 minutes) to separate the red cells from the plasma. Next, the cells were frozen at -80 °C. Each sample was taken, saponified and transmethylation was performed using a 6890 Chromatograph (Supelco, Bellefonte, Pennsylvania, USA). The results were given as a percentage of the total fatty acid composition.

### Statistical analysis

The sample size of 100 subjects (at least 50 in each group) was previously calculated to have a 90 % statistical power and effect size ≥ 0.5 and significance level α = 0.05. The categorical data are presented as counts and percentages. The continuous variables are described by means and standard deviations or by medians and ranges when the distributional assumptions were in doubt. A standard deviation greater than half of the value of the respective mean was the criterion for data normality analysis. We used the Chi-square test or Fisher’s exact test to compare the categorical data. For the initial comparison of the quantitative data, we used Student’s *t*-test to compare the means and the Mann-Whitney *U* test to compare the medians.

We conducted an adjusted comparison between smokers and nonsmokers using the analysis of covariance model (ANCOVA) including age, sex, education, body mass index (BMI), alcohol consumption and physical activity. The inclusion of factors in the ANCOVA model was based on a P-value threshold of 0.20 and an underlying conceptual framework. Fish consumption (in grams per month) was log transformed in order to minimize the impact of disruption of data normality assumptions. The data are presented as geometric means and the relative difference between current smokers and non-smokers are expressed using a measure of fold change, the geometric mean ratio (GMR) with 95 % confidence interval. The strength of the association (effect size) between smoking and fish consumption was also assessed using η^2^ (eta-square) model statistics with an interpretation scale similar to r^2^ (Pearson’s linear coefficient of determination).

The association between smoking and level of nicotine dependence with fish consumption was evaluated using Spearman’s correlation coefficient (r_S_).

The statistical significance was set at α = 0.05 and the data were analyzed using SPSS version 22.0.

### Ethical approvals

The study was approved by the Research Ethics Committee of PUCRS, Porto Alegre, Brazil under number 105.188 and by the Research Ethics Committee of the University of Toronto/University Health Network, Toronto, Canada, through opinion 12-0514BE.

## Availability of data and materials

The dataset supporting the conclusions of this article are available in the Pontificia Universidade Catolica do Rio Grande do Sul (www.pucrs.br/FAMED) or in the e-mail address of the correspondent author.
